# A unique presentation of superinfected pseudomyxoma peritonei secondary to a low-grade appendiceal mucinous neoplasm

**DOI:** 10.1186/s12957-019-1578-8

**Published:** 2019-02-18

**Authors:** Brianne J. Sullivan, Nathan Bolton, Umut Sarpel, Deepa Magge

**Affiliations:** Department of Surgical Oncology, Mount Sinai St. Luke’s West Hospital, 425 W. 59th St., 7th Floor, New York, NY 10019 USA

**Keywords:** Pseudomyxoma peritonei, PMP, LAMN, Appendix, Sepsis, Infected

## Abstract

**Background:**

Pseudomyxoma peritonei (PMP) is an uncommon condition characterized by diffuse mucinous material in the abdomen and pelvis, generally arising from a perforated epithelial neoplasm. Typically, the disease presents as suspected acute appendicitis, ovarian mass, abdominal distension, or ventral hernia. Our case represents a very rare presentation of superinfected PMP.

**Case presentation:**

A 46-year-old female with a past medical history notable for depression, asthma, and uterine leiomyomas presented to an urgent care with 5 days of progressive abdominal pain, bloating, nausea, and subjective fevers. The patient had a diffusely tender abdomen, without peritonitis, was mildly tachycardic, and had a white blood cell count of 15 K. A CT of the abdomen/pelvis was consistent with PMP with a ruptured appendiceal mucocele versus PMP secondary to an adnexal ovarian neoplastic pathology with an infectious component. The patient initially improved on antibiotics but ultimately required two surgeries, the first of which controlled intraabdominal sepsis while the second permitted definitive management of PMP with cytoreductive surgery (CRS) and HIPEC.

**Conclusion:**

Superinfected PMP is a rare entity with very few documented cases. A staged approach that incorporates clearing the peritoneal infection, with or without resection of the primary tumor, followed by rehabilitation and definitive surgery appears to be a safe and effective management strategy.

## Background

Pseudomyxoma peritonei (PMP) is an uncommon condition characterized by diffuse mucinous material in the abdomen and pelvis, along with implants on peritoneal surfaces and omentum, secondary to a perforated mucinous neoplasm [1]. The first clinical case of PMP was described in 1842 by Carl Rokitansky, but the term was coined in 1884, in association with a mucinous carcinoma of the ovary. Pseudomyxoma peritonei was described again in 1901 in association with a cyst of the appendix [1, 2]. PMP initially referred primarily to intraperitoneal mucinous spread originating from a cystadenoma of the appendix, but the term now also encompasses mucin-producing neoplasms with intraperitoneal dissemination from the appendix, small and large bowel, pancreas, lung, breast, stomach, gallbladder, fallopian tubes, and ovaries [3]. The most common presentations of PMP are suspected acute appendicitis, ovarian masses, abdominal distension, and new-onset hernia [2, 4].

Using experience at high-volume centers, the prevalence of pseudomyxoma peritonei is thought to be about three to four per million [2]. Histopathology and classification of PMP has always been challenging, with multiple classification systems and overlapping terminology. In 1995, Ronnet et al. classified PMP into three groups based upon the clinicopathologic features of 109 cases, disseminated peritoneal adenomucinosis (DPAM), peritoneal mucinous carcinomatosis (PMCA), and a hybrid type. PMCA is associated with aggressive behavior while DPAM has little histologic evidence of malignancy, is slow growing, and has a relatively good prognosis when complete cytoreduction can be achieved [5, 6]. Most recently in a consensus statement published in 2016, Carr et al. state three categories of PMP that were agreed upon—low grade, high grade, and high grade with signet ring cell. Low- and high-grade mucinous carcinoma peritonei are considered synonymous with DPAM and PMCA, respectively [2, 7]. The standard treatment involves cytoreductive surgery (CRS) followed by hyperthermic intraperitoneal chemotherapy (HIPEC). This treatment regimen is associated with high morbidity and mortality but has improved survival dramatically, with overall 5-year survival rate of 74% and overall 10-year survival rates of 63%. High-grade mucinous neoplasms may additionally be given adjuvant systemic chemotherapy [2].

Our patient had a unique presentation, with suprainfected PMP and unclear primary source. There are only two other case reports published that depict a patient presenting with infected PMP and sepsis [6, 8]. Both of those cases were treated with immediate operative intervention. Our patient appeared to initially improve with antibiotics, but ultimately also required an exploratory laparotomy and washout with staged debulking and HIPEC after the infection was controlled.

## Case presentation

This is a case report of a 46-year-old female with a past medical history notable for depression, asthma, and uterine leiomyomas who presented to an urgent care with 5 days of progressive abdominal pain, bloating, nausea, and subjective fevers. The patient endorsed a several month history of gaining weight, though she attributed it to her lifestyle, accompanied with strong, intermittent, crampy right lower quadrant pain. The pain was random in onset and would dissipate very quickly. However, 5 days prior to presentation, her pain dramatically increased and was persistent in nature.

Her surgical history included a myomectomy performed 5 years prior followed by a laparoscopic hysterectomy 2 years later. She was a regular drinker, consuming four to five alcoholic drinks per night, but stopped when her symptoms worsened and had no history of withdrawal. Family history was significant for a maternal grandmother with breast cancer, mother with skin cancer, and an uncle with colon cancer.

**Fig. 1:**
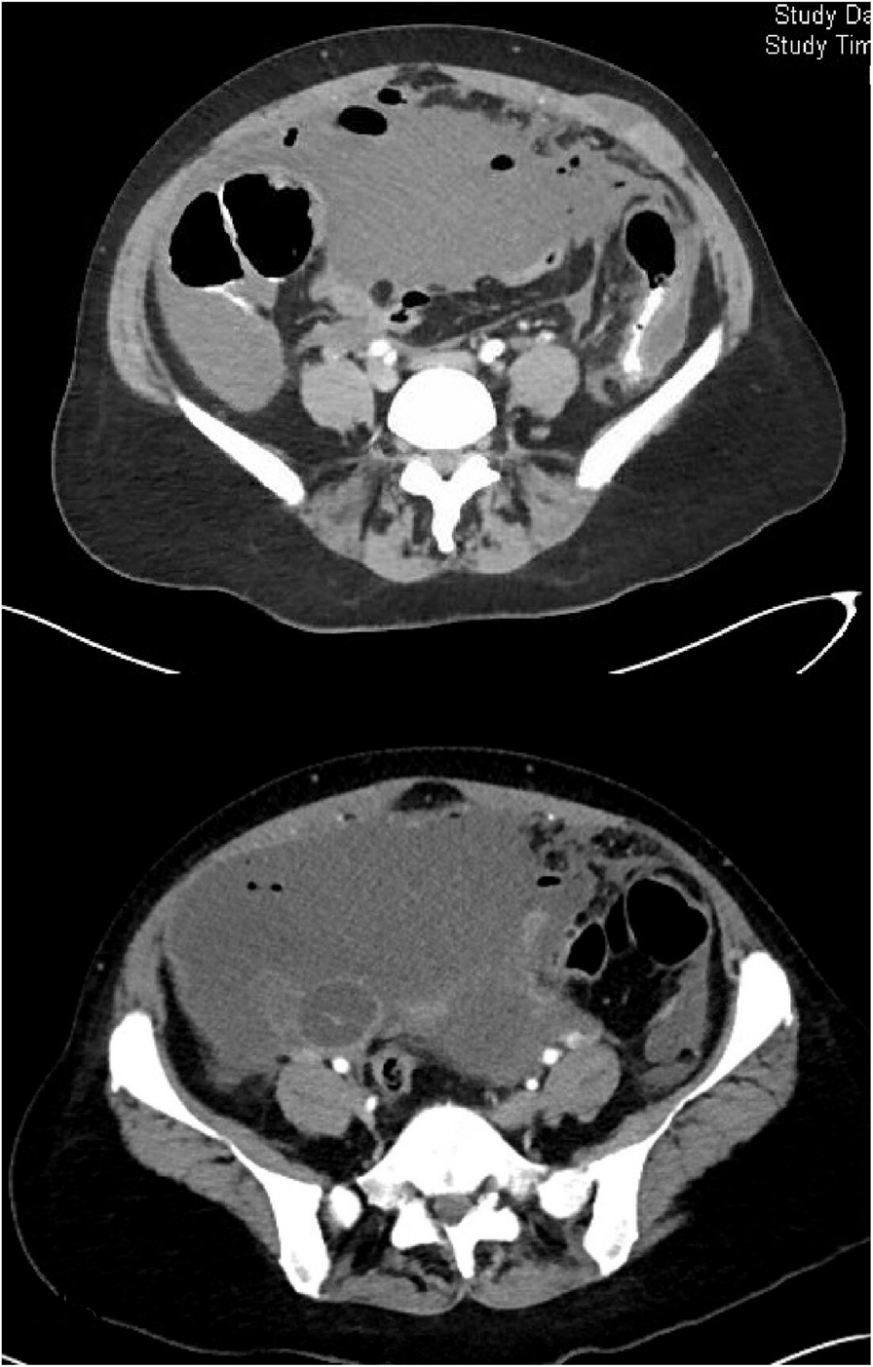
Representative images of CT of the abdomen/pelvis upon presentation with moderate-volume ascites, complex fluid within the abdomen and pelvis with extensive gas

Upon arrival to the emergency room, the patient was tachycardic, mildly hypotensive, and febrile to 103 °F. Her abdomen was soft, distended, and diffusely tender without peritonitis. Labs were notable for a leukocytosis of 15 K. CT of the abdomen and pelvis demonstrated moderate volume, complex fluid within the abdomen and pelvis with extensive amount of gas and peripheral rim enhancement, a thickened appendix filled with fluid, and a soft tissue/cystic lesion in the anterior abdominal wall (Fig. 1).

Differential included pseudomyxoma peritonei with a ruptured appendiceal mucocele versus PMP secondary to an adnexal ovarian neoplastic pathology with an infectious component. Per the radiology report, the origin was unclear based upon imaging and stated a ruptured adnexal cyst should be considered given the markedly enlarged septated cystic lesions in the pelvis. The patient was resuscitated, and her blood pressure and heart rate normalized with 2 L of fluid. Given the patient’s hemodynamic stability, the decision was made to attempt conservative management with antibiotics and interventional radiology (IR) biopsy and drainage.

**Fig. 2:**
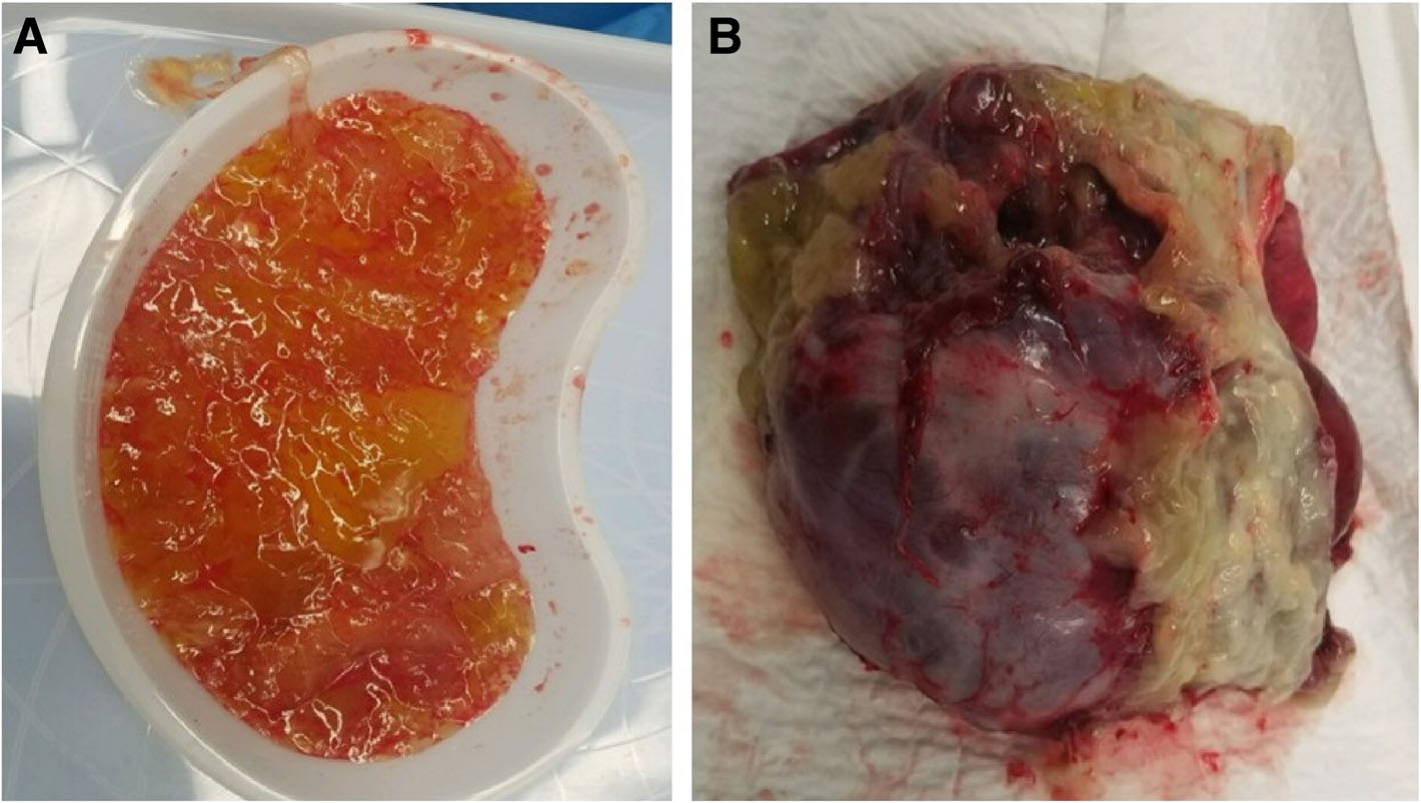
**a** Gross image of mucin removed at first operation for washout of superinfected PMP and **b** removed left ovary and ovarian cyst

On the floor, the patient remained hemodynamically stable with intermittent fevers. Her abdominal exam also remained unchanged, with persistent pain that was under control with pain medication. It was unclear if the abdominal wall mass was neoplastic, and given our initial nonoperative approach, a biopsy was performed for tissue diagnosis to properly guide further treatment. A core needle biopsy of the abdominal wall mass demonstrated a uterine leiomyoma implant, and the cytology aspirate of the peritoneal fluid showed pools of acellular mucoid material. On hospital day 7, the patient’s leukocytosis rose to 23 K, and repeat imaging demonstrated a more organized collection with intraperitoneal air in addition to multiple thick, wall-enhancing, complex cystic multiseptated lesions. Given these findings on imaging as well as her clinical presentation, the patient was taken to the operating room (OR) jointly with surgical oncology and gynecology oncology for a peritoneal washout and bilateral salpingoopherectomy. Intraoperative findings included a large amount of mucin in the abdomen and pelvis with a pocket of purulent fluid in the mid-abdomen, a very large (12 cm) cystic left ovary, dilated right fallopian tube with enlarged right ovary, a very dilated and thickened appendix, and dense, diffuse small bowel intraabdominal adhesions (Fig. 2). Her peritoneal cancer index (PCI) score was unable to be determined due to the degree of inflammatory adhesions. She underwent bilateral salpingoopherectomy given the amount of inflammation in the cecum and base of the appendix, the decision was made not to perform an appendectomy to avoid leakage at the staple line and further infectious complication. The patient’s abdomen was hostile, and no larger operation was deemed necessary at this time, as the patient was nonobstructed and the origin of PMP was not yet verified. The primary goal of the operation was to clear the infection, with the intent to return at a later date when her intraabdominal contents could be properly mobilized for a complete PCI score and an appropriate oncologic resection could be performed.

**Fig. 3:**
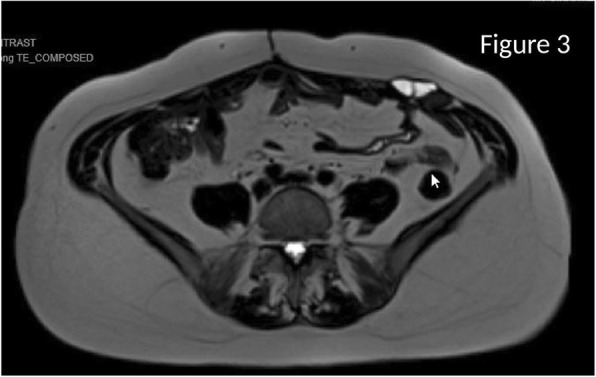
Surveillance MRI of the abdomen/pelvis with no evidence of metastatic disease

Her hospital course was complicated by recurrent pleural effusions requiring multiple thoracocenteses, negative for cytology, and persistent need for supplemental oxygen. She also developed a secondary intraabdominal abscess requiring IR-guided drainage. After the patient clinically improved, she returned to the OR 2 months after her initial presentation for completion cytoreductive surgery (CRS) and hyperthermic intraperitoneal chemotherapy (HIPEC) with 40 mg of mitomycin-C at a target temperature of 42–43 °C over 90 min, as per our institutional protocol [9]. Her PCI at the second surgery was calculated to be 13. She had minimal peritoneal adhesions, and her intraabdominal infectious process had completely resolved. The patient underwent an appendectomy, omentectomy, and tumor debulking. The appendicular base was healthy and easily stapled across. The patient was discharged home with lovenox on postoperative day 5, saturating well on room air, tolerating a diet with oral pain medication, and with return of bowel function. Six months after the CRS/HIPEC, the patient had surveillance imaging with no evidence of recurrence (Fig. 3). The abdominal wall mass was unchanged from prior imaging and, given the biopsy of leimyoma, presumed to be an implant at the port site from the patient’s prior hysterectomy.

After the discharge from the second surgery, the patient’s postoperative recovery was complicated by new-onset shortness of breath after the completion of her 30 day course of lovenox. She was diagnosed with bilateral pulmonary emboli, with no evidence of deep vein thrombus on lower extremity duplex. Therapeutic lovenox was initiated, and her symptoms gradually improved. An interval angio CT of the chest demonstrated resolution of the clot. Anticoagulation has since been discontinued, and the patient will continue with 6-month interval surveillance imaging for PMP recurrence.

**Fig. 4:**
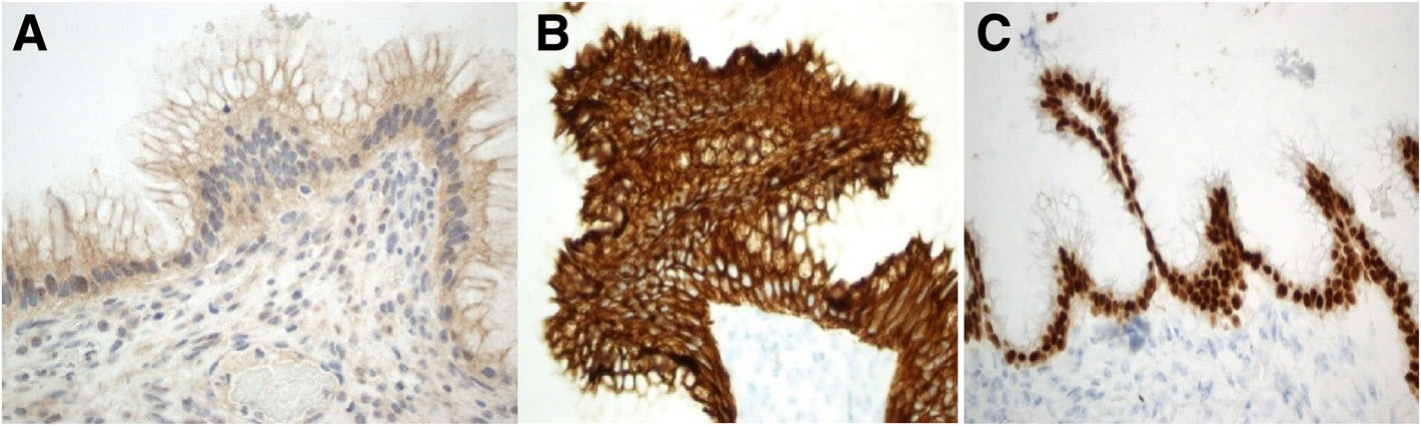
**a** Histology slide of the left ovary and tube with immunohistochemical staining negative for PAX8, **b** positive for CK7, and **c** positive for CDX2

The pathology of the left ovary and tube following the first operation resulted as an ovarian cyst containing mucin pools and low-grade intestinal type mucinous glands, consistent with metastasis from appendiceal mucinous neoplasm. The right ovary and tube showed mucin, acute inflammation, and adhesions. Immunostains were positive for CK7, CK20, and CDX2 and negative for PAX8 (Fig. 4). Following the completion cytoreduction, the appendiceal pathology demonstrated a low-grade appendiceal mucinous neoplasm (LAMN) with acellular mucin outside the appendix and associated fibroinflammatory response. The proximal margin, the appendicieal base, was negative for tumor. Additional specimens sent during the debulking included the falciform ligament, omentum, pelvic tumor nodules, and tumor deposits overlying the sigmoid, small bowel, left colon, and liver. All additional specimens consisted of acellular mucin. Pre-operative carcinoembryonic antigen (CEA) and CA-125 were elevated at 26.8 and 101 respectively. CA 19-9 was within the normal limits. Using AJCC (8th ed.) staging, the final staging was determined to be T4a NX M1a (stage IVA). Since the patient had a complete cytoreduction and the pathology was determined to be LAMN, no further adjuvant therapy was given.

## Discussion

PMP is a complex entity that can be difficult to treat due to its heterogeneity and rarity. Survival has been lengthened significantly with complete cytoreductive surgery and HIPEC [2, 10]. However, this approach has been associated with high morbidity and mortality, with mortality reaching 4.4% and major morbidity spanning from 7 to 49%, making patient selection important [2]. Nonetheless, the most important prognostic factor determining the true benefit of this definitive surgery is the ability to perform complete cytoreduction (CCR), achieving CCR-0 (CCR0 = no macroscopic disease or CCR1 = < 0.25 cm of macroscopic disease). Currently, surgery remains the best method available to improve both progression-free and overall survival in the appropriate patient population with low-grade pathology [2]. A retrospective multi-institutional review of over 2000 patients with appendiceal mucinous neoplasm treated with CRS demonstrated that prior chemotherapy treatment, no HIPEC, PMCA histopatholgic subtype, and a CCR 2 or 3 debulking surgery led to poorer prognosis [11]. Neoadjuvant chemotherapy is not typically given, and adjuvant chemotherapy can be considered for patients with high-grade pathology [2].

Our case report describes a patient with superinfected pseudomyxoma peritonei treated with initial conservative management with interval completion cytoreductive surgery once her infection was controlled. There are two other cases of superinfected PMP reported on PubMed and Google Scholar. The first was a patient diagnosed with PMP a few months after an episode of small bowel obstruction. He presented with presumed appendiceal rupture and bacterial contamination of mucus causing sepsis and peritonitis [6]. As with our patient, he required a staged surgery. The first surgery was an appendectomy and washout followed by interval cytoreductive surgery and HIPEC 1 month later. This differed from our case in that we did not resect the appendix in the first stage despite its appearance due to the risk of leak at the resection point. The second case report describes a patient with PMP of unknown etiology who presented with fever, malaise, and a palpable abdominal mass on physical exam and was found to have diffuse pyogenic ascites mixed with mucinous fluid on exploratory laparotomy. A complete cytoreductive surgery was unable to be performed, so he was placed on adjuvant intraperitoneal chemotherapy with cisplatin followed by the administration of mitomycin C and 5-fluorouracil. The patient did develop an enterocutaneous fistula, which he recovered from and survived 2 years without any major further morbidity [8].

In our patient, it was unclear whether the appendix or the ovary was the primary etiology of her peritoneal disease. PMP is two to three times more prevalent in women, and although there is evidence that the appendix is the most common source, primary ovarian-based tumors may help to explain the gender distribution. In a single-center study by Peng et al., of 35 females with PMP, 60% had appendiceal-based tumors and 34.3% had ovarian [12]. Tumor markers have been shown to have an association with prognosis. One large retrospective review included 519 patients with PMP of appendiceal origin and found that patients who had all three elevated tumor markers (CEA, CA-125, CA 19-9) had significantly shorter overall survival and higher risk of recurrence compared to those with normal tumor maker levels. Thus, tumor markers may be able to predict an increased risk of recurrence after complete CRS and guide postoperative follow-up or second look surgeries [13]. The presentation of PMP with peritonitis is very uncommon, with the most likely etiology either bacterial translocation secondary to tumor perforation or ruptured appendicitis causing bacterial contamination [6, 8]. The approach to superinfected PMP is similar to the concept of damage control for a perforated viscous—control the infection, stabilize and resuscitate the patient, and perform a definitive surgery at a later date.

## Conclusion

When superinfected PMP is highly suspected from imaging or biopsy results, surgical intervention likely should not be delayed, regardless of the lack of peritoneal signs. A staged approach that incorporates clearing the peritoneal infection, with or without resection of the primary tumor, followed by rehabilitation and definitive surgery appears to be a successful management strategy.

### Learning points

- In a patient with concurrent intraabdominal infection and psuedomyxoma peritonei, a staged approach, similar to damage control surgery, allows for a safe and appropriate oncologic resection at a later date
- Superinfected pseudomyxoma peritonei is unlikely to respond solely to antibiotics and supportive care given the diffuse nature of the disease; thus, operative intervention with the intention of clearing the infection should be an early management strategy

## Abbreviations

CCR: Complete cytoreduction
CRS: Cytoreductive surgery
DPAM: Disseminated peritoneal adenomucinosis
HIPEC: Hyperthermic intraperitoneal chemotherapy
PCI: Peritoneal cancer index
PE: Pulmonary embolus
PMCA: Peritoneal mucinous carcinomatosis
PMP: Pseudomyxoma peritonei
